# Management of meconium ileus with perforation and formation of a huge pseudocyst: a neonatal case report

**DOI:** 10.1097/RC9.0000000000000398

**Published:** 2026-03-18

**Authors:** Omair Bseiso, Anas Zahdeh, Amany Daabes, Ihssan Ghazzawi, Haneen Siam, Ahmad Abushark

**Affiliations:** a Faculty of Medicine, Hebron University, Hebron, Palestine; b Department of Pediatrics, Palestine Red Crescent Society, Hebron, Palestine; c Department of Surgery, Palestine Red Crescent Society, Hebron, Palestine

**Keywords:** case report, cystic fibrosis, meconium ileus, neonatal surgery, pseudocyst

## Abstract

**Background:**

Meconium ileus complicated by prenatal perforation and pseudocyst formation is a severe variant requiring prompt surgical management.

**Case presentation:**

A female newborn presented with severe abdominal distention, with a prenatal ultrasound showing ascites. Postnatal imaging suggested complex meconium ileus with meconium peritonitis. Laparotomy on day 2 revealed a large pseudocyst and a single perforation. The cyst was drained, adhesions released, inspissated meconium evacuated via appendiceal catheterization, and the perforation repaired. The infant recovered well, tolerated feeds, and was discharged in stable condition. Given family history, evaluation for cystic fibrosis was initiated, and enzyme/vitamin therapy started.

**Conclusion:**

Early recognition, timely surgery, and coordinated postoperative care can achieve favorable outcomes even in complex meconium ileus with prenatal perforation and pseudocyst formation. Prenatal identification of abdominal abnormalities should prompt delivery in a center with neonatal surgical capability.

## Introduction

Meconium ileus (MI) happens when a newborn’s intestines get blocked by thick, sticky meconium in the terminal ileum. Studies show that the pathophysiology is related to dehydration of the luminal content due to impaired chloride secretion via the CFTR channel, resulting in highly viscous, inspissated meconium^[^1^]^. It is closely linked to cystic fibrosis (CF) and can show up as a simple blockage or as a more complex problem that leads to volvulus, bowel atresia, or intestinal perforation. MI occurs in approximately 15% of infants with CF^[^2^]^.

If perforation occurs before birth, leaked meconium can cause meconium peritonitis and, sometimes, form a meconium pseudocyst, a rare, encapsulated mass from sterile inflammation. Affected infants often have severe abdominal swelling or trouble breathing.

This case highlights the successful management of complex MI with prenatal perforation and the formation of a large meconium pseudocyst, demonstrating that early surgical intervention, combined with postoperative supportive care, can lead to favorable outcomes even in high-risk neonates.

HIGHLIGHTS

- Complex meconium ileus with prenatal perforation can present as a large meconium pseudocyst, highlighting the importance of recognizing prenatal signs such as ascites and calcifications.
- Early surgical intervention using cyst drainage, adhesiolysis, and appendiceal catheter irrigation successfully restored bowel function and avoided the need for bowel resection.
- The favorable postoperative course and correlation with a strong family history support the clinical relevance of early cystic fibrosis evaluation and guided nutritional therapy in similar high-risk neonates

This case report has been reported in line with the SCARE 2025 criteria^[^3^]^.

## Case presentation

A 1 hour female newborn who was delivered by elective CS to mother G3P2A0 due to IUGR and parental ultrasound showing abdominal distention, gestational age 35 + 6 weeks, detailed US was done at 29 weeks and showed: moderate ascites, urinary bladder slightly distended, mild renal pelvis dilatation (right kidney 0.7 cm), at delivery she was born vigorous, crying, Apgar score 7/9, noticed to have severe abdominal distention with absent bowel sound, birth weight 2500 g, head circumference 33 cm, length 50 cm, and she was hemodynamically stable. Although prenatal ultrasonography demonstrated ascites and abdominal distension, the original prenatal images were not available for inclusion due to institutional record limitations.

**Figure 1.**
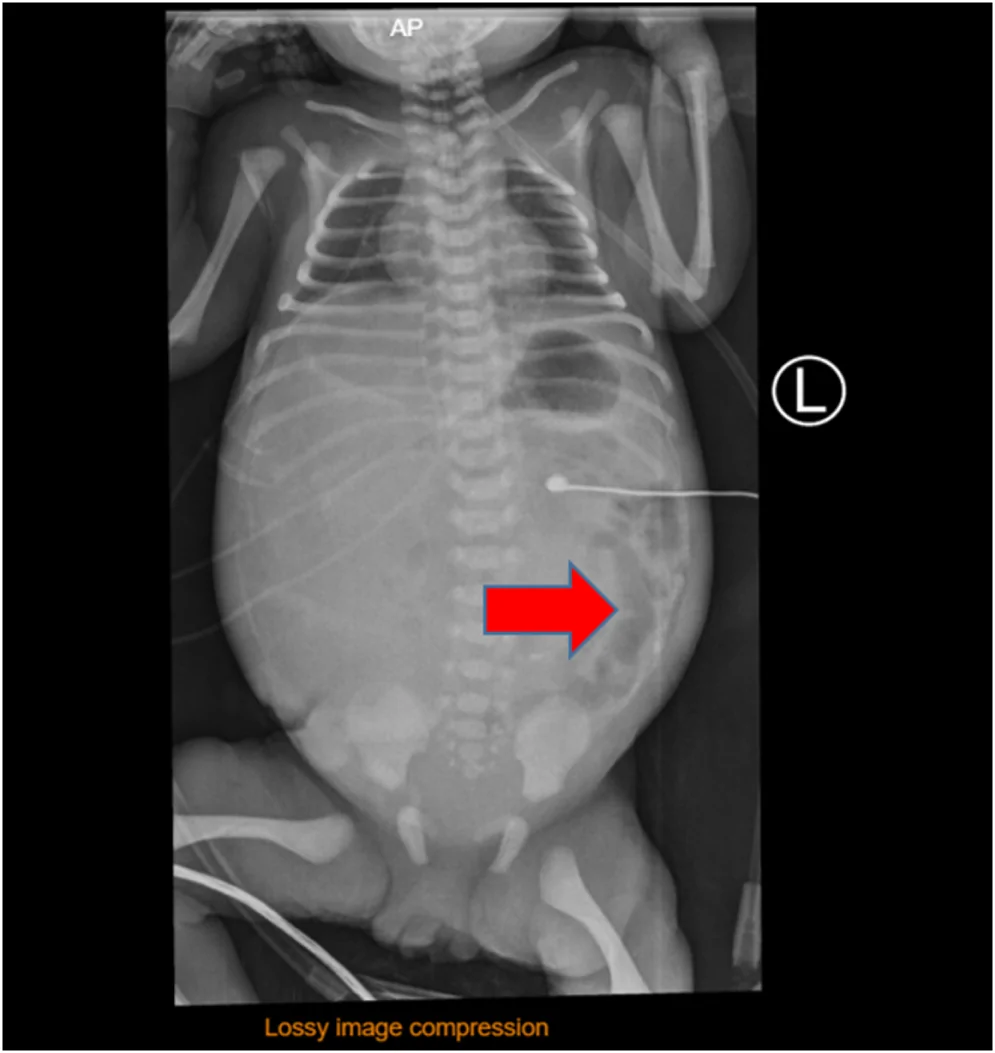
A gasless abdomen, with ground glass appearance with left-sided calcification (red arrow).

An abdominal x-ray with oral contrast was done at the age of 1 day, which showed a gasless abdomen, ground glass appearance with left-sided calcification (Fig. 1).

**Figure 2.**
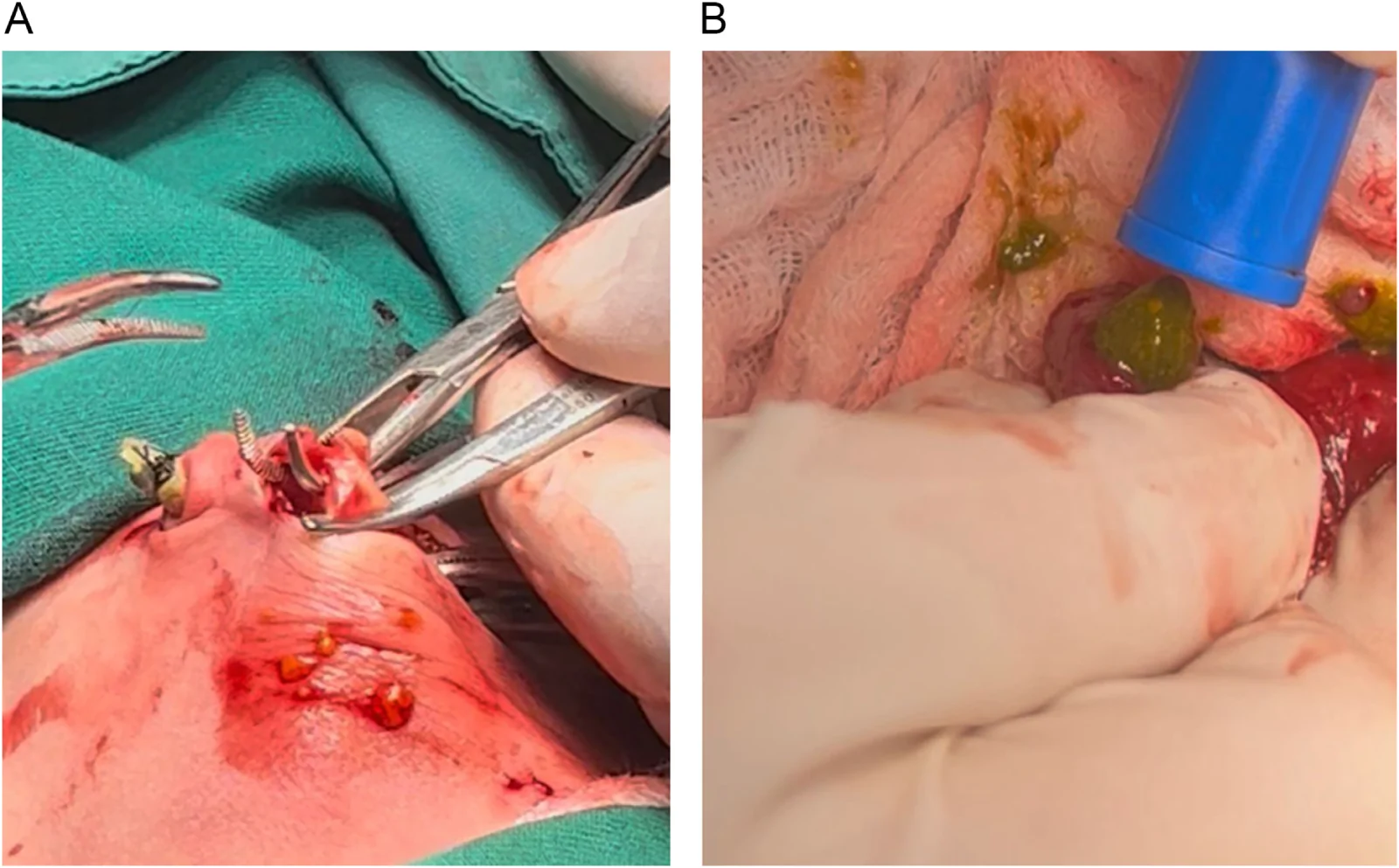
(**A**) Limited adhesiolysis was performed to mobilize accessible bowel segments. (B) Appendix opened and catheter inserted through the appendix, and the terminal ileum was irrigated, and the thick meconium evacuated.

On second day exploratory laparotomy, a large meconium pseudocyst containing thick inspissated meconium occupied a significant portion of the abdominal cavity and was adherent to the abdominal wall and adjacent bowel loops. Limited adhesiolysis was performed to mobilize the accessible bowel segments (Fig. 2A). Following evacuation of the cyst contents, the bowel was partially mobilized; however, dense inflammatory adhesions limited complete inspection of the entire intestine. A single small perforation was identified in the terminal ileum, approximately 20 cm proximal to the ileocecal valve. The bowel distal to the perforation was markedly distended and filled with thick meconium pellets. Direct irrigation through the colon was technically difficult due to the highly viscous nature of the meconium. Therefore, a catheter was inserted through the appendix to allow controlled retrograde irrigation of the terminal ileum and colon, facilitating effective evacuation of the inspissated meconium while minimizing manipulation of the perforation site (Fig. 2B). After adequate bowel decompression and irrigation, the ileal perforation was closed primarily. A diverting stoma was not created, as the bowel appeared viable with no evidence of necrosis or gangrene, and the pseudocyst represented an inflammatory covering rather than a contained sealed perforation. No bowel resection or anastomosis was required. After catheter removal, the appendix was preserved and closed.

Postoperation, the abdomen was soft and lax, extubated after full recovery, then started total fluid intake of 80cc/kg/day, and increased gradually according to protocol.

**Figure 3.**
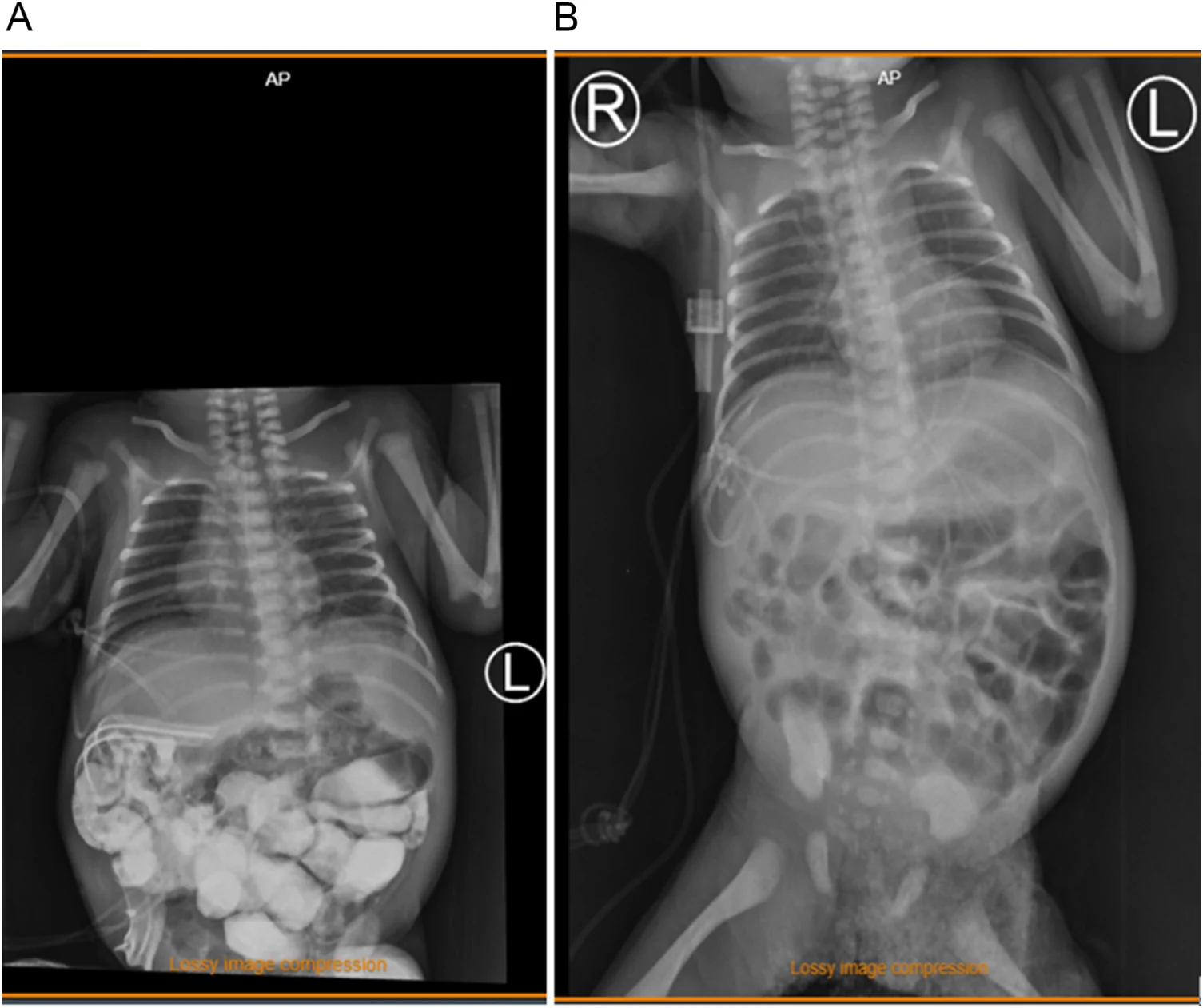
(A) No microcolon. (B) Contrast passed to the terminal ilium.

On postoperative day 12, a lower gastrointestinal study was done due to defecation difficulty, and there was no microcolon, and the contrast passed to the terminal ileum as shown in Figure 3A and 3B, and the defecation is normal now. She was kept nothing by mouth and received total parenteral nutrition for 12 days postoperatively. Then she started feeding gradually until full feeding was tolerated, with no vomiting or abdominal distention. She passed stool regularly, greenish in color.

The initial laboratory study was remarkable for an increase in WBC (21.18), HB (19.4), HTC (57.7), and platelets (442). She received one packed red blood cell after surgery. Follow-up CBC was done according to protocol. The last results were WBC (19.31), HB (10.2), and platelet (714). The coagulation profile was unremarkable.

Follow-up coagulation profile showed elevated PT, so she was given vitamin K twice. The last PT was 15.3.

CRP was 37, so she started on IV ampicillin, gentamicin, and metronidazole. On day 3 of admission, due to rising CRP (82), a new blood culture was obtained, gentamicin was stopped, and she was started on amikacin (Aminoglycoside). Follow-up CRP showed improvement; the last one was negative. All cultures came back negative.

According to her presentation and family history of CF, the patient needs genetic testing for CFTR gene (intron 9). Her sister had previously undergone genetic study, which showed genomic amplification of exon 10 of CFTR gene, and direct sequencing was performed to detect the presence of 1525-1 G>A (c.1393-1 G>A) mutation in intron 9 on the DNA extracted from a peripheral blood sample. She was found to be homozygous for the mutation 1525-1 G>A (c. 1393-1 G>A) in intron 9 of CFTR gene. The 1525-1 G>A mutation has been previously reported in CF patients as a disease-causing mutation^[^4^]^.

For our patient, as she was suspected of having CF, she was started on Pancrelipase and Vitamins (ADEK).

The patient was discharged in good condition on Pancrelipase 3000 IU with every feeding, Vitamin E 13 IU per day, and Vitamin K 0.3 mg.

## Discussion

We describe the case of a newborn that had a difficult delivery because of a large pseudocyst, a prenatal bowel perforation, and complicated MI. Defective CFTR function reduces chloride and bicarbonate secretion into the intestinal lumen, resulting in dehydration of luminal mucus and formation of inspissated meconium that predisposes to obstruction^[^5^]^. Early operation was indicated because she had marked abdominal distension and respiratory symptoms immediately after birth. Prenatal imaging findings such as ascites, bowel dilatation, and pseudocyst formation are well-established predictors of the need for postnatal surgical intervention^[^6^]^.

Meconium pseudocyst formation has also been described in association with jejunoileal atresia, midgut volvulus, intestinal ischemia, and idiopathic fetal bowel perforation. However, in this case, the presence of inspissated meconium, distal bowel obstruction, and a strong family history with a confirmed CFTR mutation in a sibling strongly supported MI related to CF as the most likely underlying etiology.

With careful surgical technique, intestinal function was preserved, and the obstruction was relieved with cyst drainage, adhesiolysis, bowel irrigation, and repair of the perforation. Management of complicated MI remains highly variable, with no universally accepted surgical protocol; reoperation rates of 18%–38% and mortality rates up to 8% have been reported^[^5^]^. Evidence suggests that earlier surgical intervention in neonates with meconium peritonitis leads to better outcomes, reducing intra-abdominal inflammation and the risk of secondary infection^[^7^]^. In our case, the decision to operate on day 2 likely mitigated further inflammatory injury and helped prevent systemic complications. The infant did well and progressed postoperatively with gradual feeds under observation.

This case serves as a reminder that if we work together, provide timely and careful care, and support these fragile infants, an excellent outcome is possible even in the most complex circumstances surrounding gastrointestinal pathology. Our case reinforces that early surgical intervention, combined with tailored postoperative nutritional and genetically based management, can result in favorable outcomes even in neonates with complicated MI. In the present case, appendiceal catheterization provided a controlled and effective route for retrograde irrigation in the setting of thick inspissated meconium, where conventional colonic irrigation was technically challenging. Primary repair without stoma was considered appropriate due to localized contamination, preserved bowel viability, and clinical stability.

## Conclusion

This case demonstrates that even highly complex presentations of MI, particularly those involving prenatal perforation and spontaneous pseudocyst formation, can achieve favorable outcomes when early recognition, timely surgical intervention, and coordinated postoperative care are provided. Prenatal findings such as ascites or cystic abdominal masses should alert clinicians to the likelihood of severe disease and the need for delivery in a center capable of immediate neonatal surgical management.

The successful outcome in our patient highlights the value of meticulous intraoperative assessment, effective evacuation of inspissated meconium, and restoration of bowel continuity in reducing postoperative morbidity. When CF is suspected, early nutritional support and genotype-guided therapy are essential components of comprehensive care.

As survival continues to improve for infants with complex MI, this case reinforces the importance of a multidisciplinary approach and sustained long-term follow-up to optimize gastrointestinal function and overall health.

## Data Availability

No new datasets were generated or analyzed for this article. All relevant data supporting the findings of this case report are included within the article.

## Research registration unique identifying number (UIN)

1. Name of the registry: Not applicable. 2. Unique identifying number or registration ID: Not applicable. 3. Hyperlink to your specific registration (must be publicly accessible and will be checked): Not applicable.
